# Technologies and Solutions for Cattle Tracking: A Review of the State of the Art

**DOI:** 10.3390/s24196486

**Published:** 2024-10-09

**Authors:** Saúl Montalván, Pablo Arcos, Pablo Sarzosa, Richard Alejandro Rocha, Sang Guun Yoo, Youbean Kim

**Affiliations:** 1 Smart Lab, Escuela Politécnica Nacional, Quito 170143, Ecuador; 2 Departamento de Informática y Ciencias de la Computación, Escuela Politécnica Nacional, Quito 170143, Ecuador; 3 Departamento de Ciencias de la Computación, Universidad de las Fuerzas Armadas ESPE, Quito 171103, Ecuador; 4 Department of Semiconductor Engineering, Myongji University, Yongin-si 17058, Republic of Korea

**Keywords:** cattle tracking, animal monitoring, location, solutions, IoT, camera, drone, Industry 4.0

## Abstract

This article presents a systematic literature review of technologies and solutions for cattle tracking and monitoring based on a comprehensive analysis of scientific articles published since 2017. The main objective of this review is to identify the current state of the art and the trends in this field, as well as to provide a guide for selecting the most suitable solution according to the user’s needs and preferences. This review covers various aspects of cattle tracking, such as the devices, sensors, power supply, wireless communication protocols, and software used to collect, process, and visualize the data. The review also compares the advantages and disadvantages of different solutions, such as collars, cameras, and drones, in terms of cost, scalability, precision, and invasiveness. The results show that there is a growing interest and innovation in livestock localization and tracking, with a focus on integrating and adapting various technologies for effective and reliable monitoring in real-world environments.

## 1. Introduction

The global demand for food is projected to increase by 70%; in parallel, meat production must increase from 41 kg at present to 52 kg by 2050 (annual meat production from 200 million to 470 million tons) [1]. Traditional livestock management techniques are deemed insufficient to meet this demand even for small-scale farmers. The importance of exploring and implementing new, innovative solutions in livestock management is paramount to address the challenges posed by such significant increases in demand and production targets. In this context, Industry 4.0 employs technology, particularly the Internet of Things (IoT), to enhance management by integrating various technologies, sensors, and actuators to monitor, control, and track livestock in real time [2]. The application of Industry 4.0 technologies to address critical issues such as cattle theft is highlighted, as technology enhances security within the livestock industry. Similarly, there has been a significant reduction in the time invested in identifying and managing individual animals, thereby enhancing operational efficiency. Livestock tracking and monitoring facilitate the recognition of abnormal patterns and behaviors, enabling more informed and proactive decision-making processes [3].

In this context, the present article aims to perform a systematic and comparative review of current technologies used in cattle tracking and monitoring. Beyond describing these technologies, our purpose is to critically analyze their characteristics, advantages, and disadvantages, and to provide practical guidance for the selection of the most appropriate solutions in different operational scenarios. By identifying the strengths and limitations of each method, we seek to guide farmers, researchers, and stakeholders in making informed decisions on the implementation of cattle tracking technologies.

Livestock tracking, which encompasses the monitoring and management of various farm animals, differs from cattle tracking, which focuses solely on monitoring cattle. Cattle tracking plays a crucial role in this scenario by monitoring and recording the movement, location, and identification of individual cattle within a herd or throughout the supply chain. Numerous methods have emerged to perform this task, including the use of intelligent collars, ear tags, biometric identifiers, and Radio Frequency Identification (RFID) technology [4]. To improve animal–device communication, cameras and drones are integrated for visual and Global Positioning System (GPS) data acquisition.

Livestock tracking can be approached from three distinct perspectives. First, from the standpoint of food safety and public health, it is essential to ensure the safety and source verification of meat products to meet consumers’ demand for safe and traceable items. Meat products can harbor various pathogens, including *E. coli*, *Salmonella*, and *Listeria monocytogenes*, emphasizing the need for proper cattle tracking to mitigate these risks and safeguard human health [5]. Secondly, from the perspective of animal health and welfare, the implementation of efficient livestock tracking systems allows farmers to monitor the individual health of animals, recognize abnormal patterns, and control diseases more effectively. This proactive approach enhances animal welfare throughout their life cycle and helps mitigate the economic losses associated with disease outbreaks [4]. Thirdly, from a security and economic standpoint, cattle tracking helps mitigate economic losses due to theft or the disappearance of animals through real-time tracking systems. By employing technologies such as IoT-based tracking devices, farmers can monitor the location of their cattle in real time, thereby preventing theft and improving the overall security of the livestock operation [6]. For example, a wireless livestock tracking system utilizing LoRaWAN networks has been proposed to assist in real-time monitoring and theft prevention of livestock [6].

Throughout this systematic review, we not only describe a variety of livestock tracking tools, including collars, drones, and cameras used in previous research, but also conduct a detailed comparative analysis of these technologies. The objective is to examine the current research landscape in this domain, focusing on the advancement of technologies used in livestock tracking, including the development of increasingly sophisticated and adaptable solutions. Aspects such as accuracy, cost, ease of implementation, and suitability to different environmental conditions are evaluated in order to determine which methods are most effective in specific scenarios. In addition, this review aims to consolidate existing knowledge and highlight the importance of integrating advanced technologies and sophisticated algorithms into livestock practices. By identifying emerging trends and gaps in current research, our study contributes to the continuous advancement of livestock management strategies and promotes the adoption of technological solutions adapted to the real needs of the sector.

The article is structured into several sections to guide the reader through the comparative analysis of cattle tracking technologies. Section 2 details the research methodology, describing the systematic review process, research questions, databases consulted, search strings used, and article inclusion and exclusion criteria. This rigorous methodological approach allows for a comprehensive review of the current landscape of technologies in livestock tracking. Section 3 presents key information organized in summary tables covering technology solutions, data acquisition devices, sensors, power sources, wireless communication protocols, visualization software, and solution implementation levels. These tables facilitate a clear comparison of the critical components involved in the various technologies.

Section 4 provides a detailed discussion and comparative analysis of previous work, complemented with figures illustrating technological trends in livestock tracking. Different solutions applied in various scenarios are compared to understand the reasons behind their choice and evaluate their advantages and disadvantages, providing an in-depth analysis of the effectiveness of each solution. Section 5 introduces the solution selection strategy adopted for livestock tracking, presenting a comparison of the technologies identified according to key characteristics such as ease of use, cost scalability, accuracy, and invasiveness. By examining these aspects, readers will understand the strengths and weaknesses of each solution, emphasizing the importance of choosing the right technology that best suits specific needs in livestock management. Finally, the conclusions summarize the most relevant findings of the systematic review, highlighting how the analysis contributes to the field and offering insights for future research and practical applications.

## 2. Research Methodology

The overarching goal of this study is to conduct a comprehensive research aimed at identifying developed systems focused on the essential function of livestock geolocalization and monitoring. With a meticulous and rigorous methodology, the present research delves into the various technologies utilized for cattle tracking, aiming to provide in-depth insights through a comparative analysis. The methodology employed in this research is rooted in the cyclic action research process [7] adapted for systematic review purposes [8]. This approach enables an examination of the current state of technologies aligned with the research objectives while fostering a dynamic process that enhances the research itself. Through this dynamic and transformative process, wherein the obtained results serve as feedback to refine current knowledge on the subject, the study endeavors to offer a comprehensive and updated perspective.

And in this regard, to carry out the research, three phases are defined, i.e., the plan, review, and results reporting phases, which are explained in detail in the next subsections.

### 2.1. Plan Phase

In this phase, general criteria are established for the initial evaluation based on the research focus. The sought technologies must demonstrate implementations of a system that addresses both the technical aspect, encompassing physical components, characteristics, and technological capabilities, and the software aspect, including algorithms, processing, detection, optimization, and other relevant aspects. Specifically, key functions and services are considered such as real-time tracking and geolocation, health and vital signs monitoring, behavior and activity pattern detection, theft and loss prevention, reproductive and feed management, data integration for advanced analytics, and real-time alerts and notifications. These functions are essential to improve efficiency, safety, and productivity in livestock management.

The inclusion of these criteria allows for the review to focus on comprehensive systems that provide practical and advanced solutions to current livestock management challenges. By clearly stating the functions and services of interest, it ensures that the articles selected are relevant and contribute to the objective of providing a detailed comparative analysis of the most effective technologies in different scenarios.

On the other hand, previous works that do not address localization or those that are not focused on livestock animals (e.g., pigs, poultry, among others) are discarded. Similarly, those that focus only on analyzing a specific part of the system, such as data security analysis or storage, without developing a comprehensive implementation or solution, are also excluded.

With this background in mind, the following research questions were formulated to guide this study:

- What technologies are utilized for cattle tracking, and which types of data are deemed relevant within this context?
- What specific functionalities and services are offered by operational livestock tracking systems?

Based on these questions, the keywords “cattle”, “livestock”, “tracking”, “geolocation”, and “localization” were identified. From these keywords, a comprehensive search string (“cattle tracking” OR “cattle geolocalization” OR “livestock tracking” OR “livestock geolocalization”) was constructed. The scientific databases selected for the literature search included ACM, IEEE, Science Direct, and Scopus, which was due to their comprehensive coverage of relevant research in fields, such as computer science, and electrical engineering, ensuring access to a diverse range of scholarly articles and publications pertinent to the research.

The meticulous construction of the search string aimed to focus the research on articles that thoroughly address the evaluation of technologies for locating livestock. This entails a comprehensive examination of both technical aspects and the associated algorithms. Additionally, a filter was applied to retrieve results from the year 2018, considering that technology becomes obsolete after five years as stated in [9].

### 2.2. Review Phase

Once the search string was defined, it was applied to each digital library (scientific database), as depicted in Table 1; also shown is the number of articles obtained from each one. The selected format for exporting the set of results was BIBTEX. These files were then utilized to import relevant information from the articles into “Rayyan”, a web-based tool designed to assist researchers conducting systematic reviews [10].

A collaborative review was established in Rayyan to enable the authors of this study to initiate the article review process. The initial step involved the elimination of duplicates with the assistance of Rayyan. As the tool displays the percentage of similarity alongside the title, duplicate articles were identified by sorting them alphabetically and examining those with identical titles and a high percentage of similarity, which were carefully reviewed and subsequently removed. The remaining documents included those with either the DOI or the URL, facilitating access to the full article.

**Figure 1:**
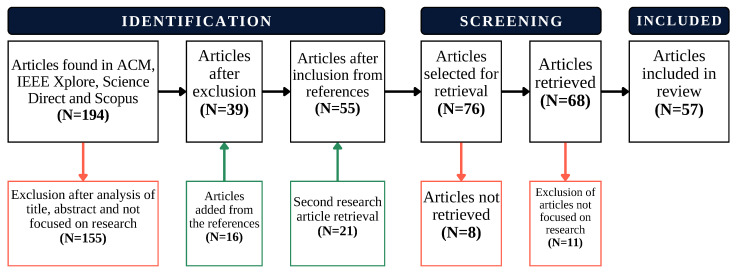
Flow of article identification and selection from databases via databases and others.

Subsequently, the authors proceeded to assign a label to each searched article using Rayyan’s labeling system, which includes three primary labels: the green “INCLUDE”, the white “MAYBE”, and the red “EXCLUDE”. The criteria for determining these labels were established during the planning phase and were customized by each author. Upon reviewing the titles and abstracts of the articles, the reasons for excluding or including an article were shared, fostering open discussions within the team to resolve conflicts and ascertain the relevance of each article to the research. Figure 1 meticulously outlines the phases undertaken for the inclusion and exclusion of articles in the review process. Using a specific color scheme, the trajectory of the review is illustrated: blue denotes the progression of the process, whereas green and red indicate the number of academic articles that were included and excluded at each stage, respectively.

Following the initial discussion, a total of 39 articles were identified for review. Recognizing a lack of papers, the authors conducted a thorough examination of the references cited in these selected articles. Subsequently, after evaluating the titles and abstracts of these references, 16 additional articles were incorporated into the review process. Furthermore, alternative search strings incorporating keywords such as “CATTLE”, “TRACKING”, “GPS”, “GEOLOCATION”, “TRACKING”, and “LOCALIZATION” were employed, resulting in the inclusion of 21 additional articles. Due to the shortage of articles, the timeframe was changed to 6 years, and some relevant articles from 2017 were considered. Ultimately, the total number of articles reached 68, as challenges were encountered in obtaining 8 of the initially selected documents.

### 2.3. Results Reporting Phase

During this phase, a meticulous review of the selected articles was conducted, focusing on extracting relevant information pertaining to technologies, solutions, protocols, and devices. Additionally, within this phase, 11 documents were identified that deviated from the primary objective of the research. Some of these documents explored technologies unrelated to the scope of this survey, while others delineated solutions applicable to animals not pertinent to livestock. Finally, a total of 57 articles were reviewed.

## 3. Cattle Tracking Solutions

The available solutions for cattle tracking were systematically examined, with a particular focus on localization methods and the diverse range of technologies utilized to achieve this goal. This systematic review does not focus on a specific technology or mechanism, but rather aims to identify optimal solutions applied in the various stages of cattle tracking, which include animal or herd detection; assigning a unique identifier to each animal, group, or breed; and, finally, tracking their movement. This detailed analysis contributes significantly to the advancement of cattle management, providing a synthesis of the different technologies used, considering each use case. Furthermore, when the article reference number is repeated in two categories, it indicates a hybrid solution.

### 3.1. General Perspective of Solutions

This is the main subsection where solutions are categorized based on the gathered information. The four main categories of solutions correspond to drones, cameras, collars, and a category for others, as presented in Table 2, with the number of articles employing each solution.

#### 3.1.1. Collar

The collar is an accessory fastened around a cow’s neck that allows the attachment of casings containing one or more devices inside. As shown in Table 1, it is the most widely used solution, with a total of 36 articles ranging from commercial collars to designs and implementations suitable for avoiding causing harm or discomfort to the animal. In [13], the authors designed a device considering aspects such as size, weight, and fasten method; the collar was manufactured using tape and plastic buckles, which can be adjusted with velcro.

Similarly, in [23], a case was 3D-printed, and all the sensors implemented to measure the animal’s parameters were placed in the case. A box for the system components was 3D-modeled and used a solar panel to recharge the battery, intended to place the GPS antenna and the temperature sensor [25]. Another way to use the collar is described in [14], where the authors placed GPS devices inside a pouch to improve water resistance and avoid unwanted button presses. In [24], authors attached tags to the cow collars using a belt. And in [19,33,35,36,41,42,43,45], the authors manufactured a case to protect commercial devices as dataloggers and GPS units.

The use of commercial collars was employed by the authors in [21,29,40,43]. In [21], the authors select the collar manufactured by ReachFar considering its economic feasibility and its ability to be charged by sunlight. In [29], an automated virtual fencing collar eShepherd was used, which contains GPS technology to control cattle movement by audio cues. In [40], the authors used a virtual fencing collar manufactured by Vence Corp, which includes devices for communication, localization, and actuators to give electrical stimuli to the animal. In [43], Lotek 3300LR GPS collars were used, placed on steers within paddocks and in the herd.

One of the advantages of using collars is that they do not only track the animals but can also be used for data collection, as mentioned in [31]. The authors collected vital signs of cows to address health problems in livestock due to the lack of effective supervision in pastoral areas. To enhance dairy cow performance, it is essential to supply adequate nutrition and maintain their welfare. However, the industry faces challenges due to diseases, lower insemination rates, and reproductive health issues. These factors contribute to reduced milk production, early exclusion of potential cows, and fewer calves. Diseases such as Foot and Mouth Disease (FMD), Anthrax, Black-quarter, and Bovine mastitis also affect cattle.

The use of body sensors and wearable technologies in animal healthcare systems is gaining prominence, as wireless sensor networks allow for the periodic monitoring of cattle activities, collecting data on nutrition, reproduction, and health parameters. In particular, the use of smart collars becomes an enabling technology that allows for the direct integration of these sensors into the animals, thus facilitating the effective implementation of advanced animal health systems. In the following sections, we further explore these sensors and their contribution to the improvement of health management in animal husbandry.

#### 3.1.2. Camera

Another solution involves using cameras to identify individuals or groups of livestock with high precision, through algorithms that directly intervene in the livestock recognition process (e.g., deep learning algorithms, convolution neural networks), or provide support to configure the position of a drone or camera. One of the convolutional neural network (CNN) algorithms is YOLO (You Only Look Once) and its different versions; it was used by the authors in [46,47,49,53,54,55,56,57]. Particularly in [57], YOLO was used for the detection stage, and for the subsequent phases, an algorithm based on deep learning was developed.

It is important to mention that there are articles in which more than one solution is used. In [12], a hybrid solution is presented that combines the use of a collar and camera to detect certain diseases or anomalies in the cows. The collar sends GPS information to a gateway configured on a Raspberry Pi, which processes the cow’s position and reconfigures the camera to move and target the cow.

#### 3.1.3. Drone

A drone or Unmanned Aerial Vehicle (UAV) is an aircraft capable of being remotely controlled without a mounted pilot [66]. In [49], drones were used to recognize livestock using a camera, and GPS information was obtained through a module and sent via a cell phone. The authors in [52] mention that it is common to use active sensors to monitor the position of the animals, but this becomes expensive when there are many animals due to the need to purchase a sensor for each one. Therefore, they propose using a drone with a camera, employing a thresholding method to recognize and track the animals. Similar works are reported in [54,55,56].

In [21], the authors used drones to move animals to specific locations using a model of quadcopter drones. They emphasize using sounds to encourage the animals to move to a certain area. Similarly, in [50], an operator remotely controlled the drone and used a support camera to locate the livestock. In [27], a simulation was performed in which animals were assumed to have collars, and drones were deployed that swept the area, obtaining the specific location of each animal transmitted by the collars.

#### 3.1.4. Other

The other subsection corresponds to various solutions such as the backpack developed by the authors in [60], containing GPS devices. This backpack was used to optimize the performance of the device and improve the comfort of the animal. In [61], they were placed on the animal’s back, and a collar was also used. Similarly, in [18], the sensors were placed around the cow’s body, suitable for each parameter that needs to measure milk production through conductivity.

The use of technologies such as RFID has relevance when used in conjunction with another solution that allows for a large group of cattle to be read quickly. The prototype developed by the authors in [58] implements a drone with an RFID reader, which approaches the groups of cows and reads the RFID tags; the RFID reader, GPS, and other sensors are controlled by Arduino. In [63], a smart tag that sends data from various sensors was manufactured, and in [64], the authors placed a smart tag on the ear of each animal.

### 3.2. Device Used for Data Gathering

One of the major problems of telemetry in animals is precisely its collectability and accuracy, as mentioned in [4], mainly due to factors related to the animal itself, environmental conditions, and the technical limitations of the devices that collect the data. For this reason, the device that reads, digitizes, and processes the information collected directly from the sensors is discussed below.

This section classifies the electronic devices used in each of the analyzed articles. The Table 3 presents a summary of the devices divided into four categories corresponding to Custom, Arduino, ESP family, and Commercial.

#### 3.2.1. Custom

The first category is “Custom”, which corresponds to boards that were designed and implemented by the authors, selecting the microcontroller, sensors, and actuators specific to their application. The creation of a board that integrates all electronic components has advantages as described by the authors in [25]. The device was developed based on specific requirements for software support, size, integration of the processor with the communication module, compatibility with the indoor GPS module, and power consumption. For this purpose, a Pycom Expansion Board V3 CPU was selected. Similarly, in [26], three controllers were tested, of which the CubeCell AB02S from HelTec Automation was selected. This board features integrated GPS and LoRa communication modules, and only an external GPS antenna was added for faster data collection.

In [62], the goal was to locate an animal by attaching a radio frequency transmitter to it and triangulating its position using the signal strength of the received radio waves. The microcontroller selected was PIC18F26K22 from Microchip Technology, and this selection was based on several factors. First, its 64 MHz clock frequency, which allows it to measure the propagation time of the radio signal with a resolution of 5 m and, the second, its low power consumption that reaches 350 nA in sleep mode. In addition, a Murata CMWX1ZZABZ-078 board integrating the STM32L082 microcontroller and the SX1276 LoRa module was selected in [28]. Similarly, authors in [32] employed the Adafruit Feather M0 RFM95 board, which features a built-in LoRa module.

On the other hand, ref. [63] used a Time-of-Arrival (ToA) triangulation method using beacon nodes placed at known locations that sends signals to a tag node placed on the animal. The beacon and tag nodes were built on a board with the dsPIC30F4013 microcontroller and the nRF24L01+ radio frequency module. Lastly, refs. [39,65] do not specify the models of the controllers used, but it is explained that they exhibit low and ultra-low power consumption, respectively.

#### 3.2.2. Arduino

The “Arduino” category corresponds to electronic devices that use or are based on development boards from the Arduino family (e.g., Arduino UNO, Arduino MEGA, Arduino Nano). In [58], the Arduino controller manages the RFID reader, GPS module, and LoRa module; it is intended to be mounted on a drone. In [23], a Pololu A-Star 328PB board based on the ATmega328PB AVR microcontroller was used; in addition, a temperature sensor and an accelerometer were integrated.

#### 3.2.3. ESP Family

The “ESPx” or ESP family category corresponds to electronic devices based on ESP32 or ESP8266 development boards. In [17], the authors developed a prototype collar using the Lilygo ESP32 board, which integrates a sim800L module for connecting to GSM/GPRS networks and uploading data to the cloud. In [20], an ESP8266 device was used as the core of the node. This node is complemented by sensors that calculate location via GPS, temperature, and humidity. In addition, the board is equipped with a LoRaWAN module (LoRa 1278) that transmits data to a Raspberry Pi-based gateway. In turn, the Raspberry Pi device sends the data to the cloud for further processing.

#### 3.2.4. Commercial

The “Commercial” category corresponds to the use of industrially created and commercially available devices. The most common device is the datalogger that records the position by means of a GPS and registers it in its memory to be later processed. In [60], a GT-730FL-S GPS was placed inside the cow’s backpack. Similarly, in [14], two types of GPS were equipped: i-gotU GT-600 and iTrail Logger H6000. Additionally, in [35], a Garmin Etrex 30 GPS logger was used and placed inside an assembled carcass attached to the neck of a cow.

In addition, in [33], two datalogger models were employed (Knight GPS and Lotek GPS 3300). It is noted that these devices have a power saving mode, but the authors suggested that this is unnecessary, and most likely, the datalogger’s memory will be filled before the battery is depleted. Finally, they concluded that the cost savings from the Knight GPS collars could help range managers make better decisions about livestock use in their grazing areas.

Another alternative consists of commercially available collars designed for continuous monitoring of livestock such as the RF-V26 model manufactured by ReachFar. This device was used in [21] for its economic feasibility and solar recharging capability. In [19], a Cluster Geolocation System 500 series was used to locate each mobile sensor through triangulation by measuring the time of arrival of periodic messages.

### 3.3. Sensors

The importance of sensor and biosensor analysis lies in their ability for early detection and prevention of disease. Sensors such as accelerometers, gyroscopes, and biosensors can collect detailed information about the behavior and physiological parameters of animals. Authors in [68] demonstrated that three-dimensional accelerometers can be used to recognize behavioral patterns in cows, making it possible to identify changes associated with disease or stress. By monitoring variables such as rumination, resting time, and general activity, it is possible to detect abnormalities that indicate health problems before they become clinically apparent. Biosensors can measure biochemical indicators, such as cortisol or glucose levels, providing valuable information about the animal’s immune and metabolic status [69]. These sensor technologies not only improve early detection of disease, but also contribute to prevention by continuously monitoring risk factors and environmental conditions.

The research methodology section mentions that one of the goals of the present work is to identify what kind of data are retrieved to monitor the animal’s health, behavior, location, and estrus and lactation periods. In this aspect, Table 4 is divided as follows:

- **GPS:** Electronic device used for obtaining the geolocation data of the node.
- **Humidity:** Sensor used to measure the humidity present in the environment or in a specific material.
- **Temperature:** Sensor used to measure ambient or animal’s temperature.
- **Pulse:** Sensor used to measure an animal’s heart rate.
- **Accelerometer:** Sensor used to measure the lineal acceleration of an object.
- **Other:** Variety of sensors to measure gas parameters (MQ2), respiration, movement and inclination (gyroscope), pH (potential of hydrogen), distances (ultrasonic), steps (pedometer), sound generators (passive buzzer), and vibration (motor).

In [17], the authors implemented temperature, humidity, and heartbeat sensors, which were used to monitor the health status of the cattle and detect any signs of illness or stress. The temperature sensor measures the body temperature of the cattle, an indicator of a fever or an infection, and the normal range of temperature for cattle is between 37.78 °C and 39.17 °C according to the above-mentioned article. Furthermore, the humidity sensor measures the humidity level of the environment, which affects the comfort and productivity of the cattle. The heartbeat sensor reflects their physical and emotional states. The article established that the normal range of heartbeat for cattle is between 100 and 140 beats per minute. Changes in heartbeat can indicate pain, fear, excitement, or disease. Finally, the collar use a respiration sensor that was classified in the category “Other”, which is one of the indicators of cattle welfare.

The use of accelerometers is common when the solution of the proposed research is a drone, as in the case of [49] because it is necessary to control the speed and the movement of this device. However, this kind of sensor can also be used in collars as in the case of [18]. In this article, the authors explain that the accelerometer measures the cattle’s activity level, which is an indicator of their health status. For example, a decrease in the activity level may suggest that the cattle is sick, injured, or pregnant. The accelerometer can also measure the eating and ruminating behavior of the cow by detecting the movements of the nose and jaw. The frequency and duration of these behaviors can reflect the cattle’s nutritional and digestive health.

Within the “Other” category are articles that utilize sensors that are not commonly employed in this context. These include a respiration sensor, as previously discussed in [17], as well as ultrasonic sensors [67], tilt sensors [15], MQ2 gas sensors [18], illumination sensors, CO_2_ and NH_3_ sensors [32], and even a pedometer [34] for counting animal steps.

### 3.4. Power Supply

The power supply of the devices that accomplish the function of tracking and monitoring of animals is vital. This characteristic can make the solution more attractive to the users, depending on whether they must constantly recharge the devices or not. The rechargeable battery applies only to solutions using collars, drones, and, specifically, to solutions that are not directly connected to a constant power source, such as the power outlet. The solution found is to use rechargeable batteries (e.g., Li-Po, Li-Ion batteries). Some studies used a solar panel as a backup to avoid disconnecting the device from the animal; this trend is summarized in Table 5. For example, in [13], a 3400 mAh, 3.7 V Li-Ion 18650 battery and a linear Li-Ion TP4056 battery charger in conjunction with a rigid solar panel (4.5 V, 0.5 W) and two flexible solar panels (2 V and 0.5 W).

### 3.5. Wireless Communication Technology

Cattle are constantly on the move, so solutions using wireless communications are needed to send the collected information. There are several wireless technologies and protocols for transporting information that connect nodes to a gateway that can be accessed from anywhere through mobile devices or computers. As presented in Table 6, the solutions were classified into six categories: LoRaWAN, Zigbee, Sigfox, Cellular, Wi-Fi, and “Others”.

In the IoT paradigm, LPWANs have emerged as a wireless communication standard with technologies that allow for covering large areas (e.g., rural areas and farms) and sending very small packets with ultra-low power consumption [70]. Standards such as LoRaWAN and Sigfox are suitable for operation in livestock environments. To comprehend Long Range Wide Area Networks (LoRaWANs), it is essential to grasp LoRa technology. Long Range (LoRa) operates as a physical layer technology within ISM bands (unlicensed sub-Gigahertz radio bands for industrial, scientific, and medical), employing a Chirp Spread Spectrum (CSS) modulation technique that allows for a range of five kilometers in urban areas and up to 15 km or more in rural areas [71]. In contrast to LoRaWAN, Sigfox is a proprietary ultra-narrowband LPWAN technology that utilizes a slow modulation rate to achieve longer ranges [70].

In [59], a microchip with LoRaWAN DV164140-2 in conjunction with a base station achieves communication between 10 km and 15 km. The prototype built in [61] uses an ES920LR device that connects to a built-in base station through LoRa. The communication distance ranges from 0 m to 2 km. In [26], the CubeCell AB01 and AB02, with LoRa transceivers, were used as end devices placed on the animal. The end nodes were connected to a LoRaWAN Gateway built using a Raspberry Pi 4 and a PG1301 shield from Dragino with a maximum distance of 8 km.

Technologies like Wi-Fi (IEEE 802.11) and Zigbee (IEEE 802.15.4) are used for applications in short-range environments [70]. Wi-Fi often proves inadequate and fails to meet expectations, particularly when considering the high energy cost it involves. In [50], a Wi-Fi network was configured in the ground control station for subsequent communication with an aircraft. On the other hand, in [61], a Zigbee Network was configured at the gateway to communicate with a celullar network and subsequently relayed to the cloud. Cellular networks have an already established infrastructure and offer great coverage in urban cities and in parts of the rural areas. In [11], it is proposed that the PASS system uses end devices that are capable of connecting to the second- and third-generation network infrastructure, specifically GSM and WCDMA, which is commonly known as UMTS. Similarly, in [17], it is mentioned that the SIM800L module allows for the integration of a SIM card to connect an ESP32 to second-generation networks (GSM/GPRS) using a data plan provided by the operator that owns the SIM card.

The category of “Others” considers articles such as [49], in which researchers use the drone’s own wireless communication to transfer the data to the cellular, and this is the one that uploads the data to a server, or radio frequency-based communications. ISM radio bands are also used with communication systems such as radio transceivers. In [62], the MRF49XA transceiver from Microchip Technology, Inc. illustrates the usefulness of an analog output, where the voltage level is related to the power of the received radio signal. In [63], the nRF24L01+ unit was used due to its characteristics of different operating speeds (250 Kbps, 1 Mbps, and 2 Mbps) and the ultra-low power consumption of the module. In [63,64], transceivers working at 2.4GHz ISM frequencies were used.

### 3.6. Software to Visualize the Information

The software used to visualize the information by the previous works can be divided into four categories: “Web application”, “Mobile applications”, “Desktop application”, and “Others”. Studies that developed and implemented these solutions are shown in Table 7. It is important to indicate that having a simple and accessible data visualization software is fundamental for farmers because it provides the necessary information for livestock management. In this context, the categories of “Web Application” and “Mobile Application” are the most popular, which consist of dynamic web pages and applications installed on mobiles devices such as smartphones and tablets. The category “Desktop App” corresponds to computer-installable software provided by commercial solutions ([33,60]) or developed by the authors ([59]).

The “Other” category corresponds to studies that used another method to send the information to the end user. In [11], a system that provides tracking through sensors, data transport and storage, and user management called Pastoral Animals Shepherd System (PASS) was developed. In this system, the user must call a mobile service code, and the system immediately responds by voice with a menu with available system options. In the other case, when the system is initiated by an event, PASS sends a message whose content is a code indicating a specific event known and detailed in a glossary. In [30,62], an API was developed that has the potential for an application over the internet.

### 3.7. Level of Implementation

This section organizes the analyzed previous works by classifying them depending on the kind of experimentation performed with the proposed solution. As presented in Table 8, the articles are divided into three categories: Simulated, Real, and Untested.

One example of the “Real” category is detailed in [50]. In this study, the proposed solution was tested in an open field, and the animals were tracked without any human intervention but only with the help of a drone. Similarly, in [29], cattle were individually trained to walk to a haystack in each paddock without any virtual fence set up. They were then collared and tested in each paddock with a virtual fence configured across the width of the paddock; testing lasted three days.

On the other hand, the “Simulated” category experimentation includes works like [6]. In this article, the network simulator NS-3 was used to create a LoRa module and simulate a scenario with one gateway and up to 10,000 end devices. Additionally, the performance of different network access techniques was compared: Time Division Multiple Access (TDMA), Carrier Sense Multiple Access (CSMA), and CSMA-10 in terms of packet delivery ratio, collision ratio, and energy consumption. CSMA is a protocol used in networks where multiple devices share the same communication channel, and it works by participating devices checking if the channel is clear before transmitting data. This helps to avoid collisions, where two or more devices can send data simultaneously, resulting in interference and data loss. LoRaWAN, on the other hand, natively uses TDMA that spreads its transmissions across different frequencies and time slots; it is concluded through network simulations that CSMA-10 and CSMA technique have better performance. Lastly, the studies in the “Untested” category only designed the system or created the prototype but did not proceed to a real environment to test their functionality.

## 4. Discussion

### 4.1. Published Articles on Cattle Tracking Since 2017

**Figure 2:**
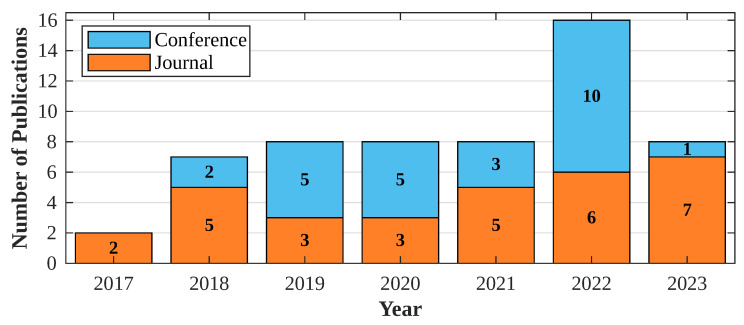
Evolution of research interest over time: number of articles published per year.

In recent years, there has been a noticeable increase in the publication of articles focused on cattle tracking and monitoring, as illustrated in Figure 2. The figure depicts a consistent annual surge in interest in developing solutions for cattle tracking, with the exception of a dip in 2020 and 2021, potentially influenced by the pandemic’s impact on research activities. However, as this article collection was conducted in mid-2023, definitive conclusions about diminished interest compared to 2022 cannot be confidently asserted.

Another important trend shown in Figure 2 is the number of conference and journal papers per year, the latter being more developed solutions at the design, implementation, and experimental levels, and, therefore, more relevant. The majority of the articles found for 2022 correspond to conferences.

### 4.2. Articles by Region

**Figure 3:**
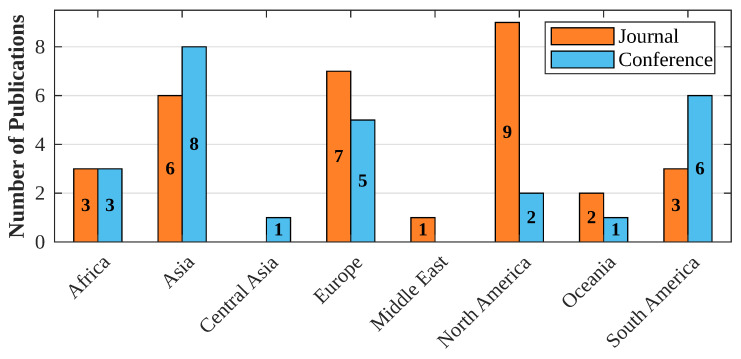
Number of articles by region and type of publication.

The trend of scientific interest in the development of livestock tracking solutions varies significantly between geographic regions, reflecting the specific needs, priorities, and resources of each area. As shown in Figure 3, North America leads in scientific journal article publications (8 articles), while Asia shows a balance between conference articles (8) and journals (6), and Europe shows considerable activity in both types of publications.

These regional differences can be attributed to several factors:

- **Technological Development and Economic Resources:** Regions such as North America and Europe have greater economic resources and advanced technological infrastructures, facilitating research and development in innovative solutions for livestock tracking. Investment in agricultural technology is a priority in these regions, driving the production of high-level research published in scientific journals.
- **Specific Agricultural and Livestock Needs:** In Asia, the growing demand for livestock products due to population growth has spurred research into efficient methods of livestock tracking and management. The balance of conference and journal publications indicates a dynamic academic community sharing advances in both international forums and formal publications.
- **Specific Agricultural and Livestock Needs:** In Asia (China, Japan, etc.), the growing demand for livestock products due to population growth has spurred research into efficient methods of livestock monitoring and management. The balance of conference and journal publications indicates a dynamic academic community sharing advances in both international forums and formal publications.
- **Regional Limitations and Opportunities:** In regions such as Africa and South America, although there is interest in the topic, the number of publications is lower. This may be due to limitations in financing, access to advanced technologies, preference for the use of traditional practices, and less visibility on international platforms. However, the interest in conference papers suggests a growing participation in global discussions and an opportunity to expand research in these areas.
- **Cultural Factors and Agricultural Policies:** Government policies and cultural practices also influence the approach to livestock monitoring. For example, countries with heavily livestock-based economies may prioritize research that optimizes production and improves animal health, while others may focus on different agricultural sectors.

### 4.3. Percentage of Solutions Used

**Figure 4:**
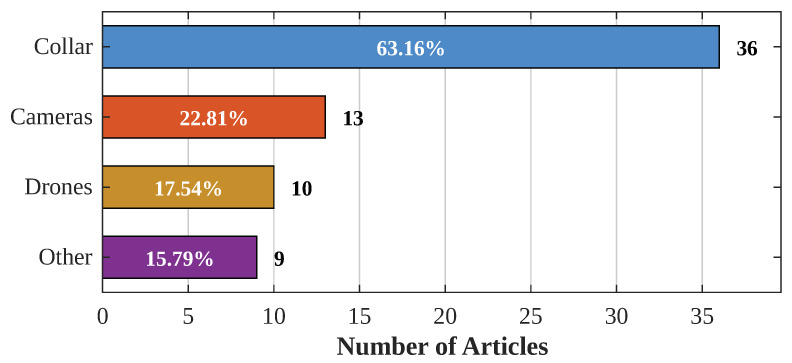
Percentage of usage by solution category.

Upon analyzing the articles, a predominant trend is the proposal of a “Collar” solution in the majority (63.16%) of cases, as indicated in Figure 4. The percentages for various devices are detailed in the same figure, with prominent use of drones as the least utilized devices for livestock tracking and monitoring. It should be noted that some solutions use more than one of the mentioned technologies, which is why the percentages do not sum to 100%. The prevalence of collars can be attributed to their ease of implementation and use in livestock, offering greater control through the integration of various sensors. However, the cost associated with collars, requiring one device per animal, increases as the size of the livestock grows.

On the other hand, the use of drones and cameras is observed to be more economical compared to collars. A single camera or drone can monitor multiple animals simultaneously, contributing to greater cost efficiency. It has been noted that static cameras cannot cover extensive areas; therefore, it is usually used for tracking animals within a predetermined area.

Concerning the use of drones, it is emphasized that their operation involves a higher level of sophistication. Although drones have improved in terms of user-friendliness, handling them in rural environments could pose a challenge. Additionally, specific knowledge about drones is necessary, and they must be operated in a way that accurately identifies animals through machine learning algorithms.

Finally, with respect to the “other” section, the use of devices categorized within this class is not recommended. This recommendation is based on the lack of concrete evidence to support the efficacy of these devices in livestock monitoring. Therefore, the study advocates focusing on collars, drones, and cameras due to their proven efficacy and practicality in livestock tracking.

### 4.4. Level of Implementation

**Figure 5:**
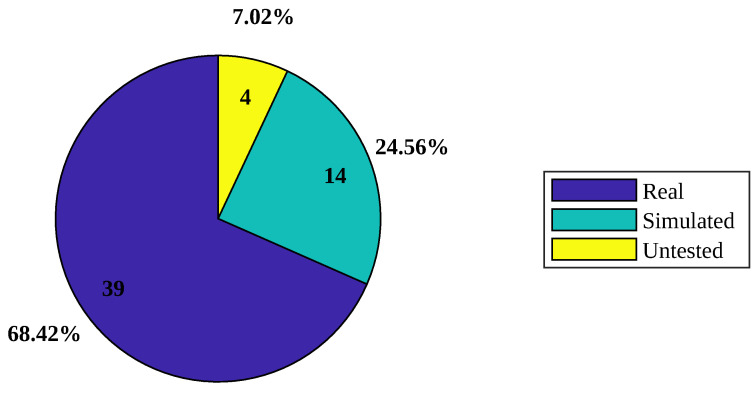
Percentage distribution of level of implementation.

The Figure 5 illustrates the percentages representing the implementation levels of the solutions. The graph highlights a noteworthy finding that 68.42% of the reviewed articles underwent testing in real-world environments. This preference for real-world experiments is justified by the invaluable ability to assess whether the proposed solutions meet the stated requirements.

However, caution should be exercised when considering the implementation of the proposed solutions; applying a solution without having previously tested it in a real environment may not be in line with the objectives. For articles that present real solutions but in controlled environments, additional testing is strongly recommended.

### 4.5. Wireless Communication Protocols

**Figure 6:**
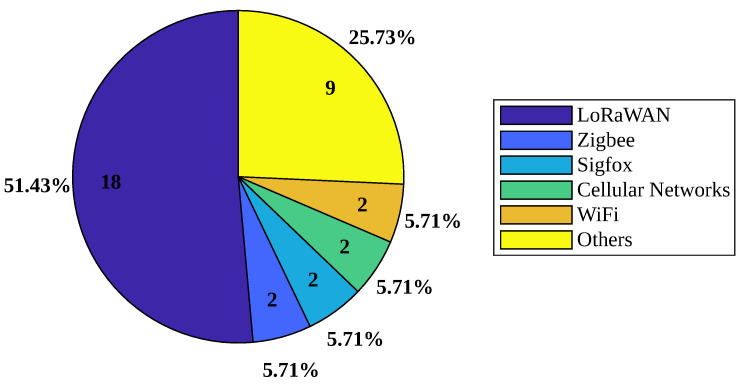
Number of articles by types of wireless communication technologies.

Authors have utilized various wireless protocols for data exchange between devices as part of livestock tracking solutions. Figure 6 shows the protocols used in the analyzed articles that use wireless communication technologies (35 articles), highlighting LoRaWAN technology as the most employed protocol (50%) for communication between implemented devices. LoRaWAN is recognized for its long signal range, which makes it an ideal wireless communication protocol for livestock tracking.

Furthermore, the prevalence of LoRaWAN can be attributed to its low power consumption, ensuring an extended battery life compared to other protocols. The analysis infers that Wi-Fi is not advisable for livestock tracking and tracing due to its limited range, suitable only for small environments.

### 4.6. End-User Visualization Applications

**Figure 7:**
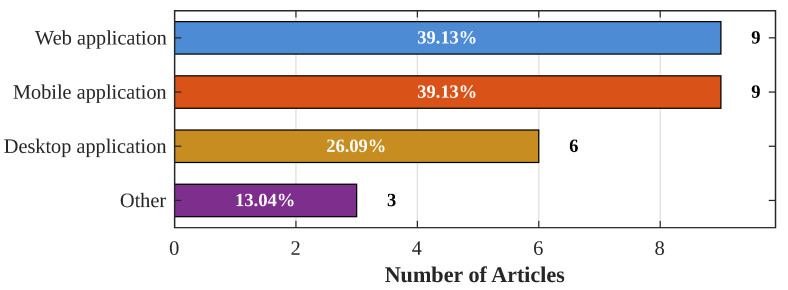
Number of articles by visualization application type, i.e., Web, Mobile, Desktop and Others.

In Figure 7, the detailed breakdown of articles incorporating data visualization applications alongside livestock tracking solutions is presented. It is also important to note that the percentages in the chart may exceed 100% because several solutions incorporate more than one type of application.

When examining the chart data, it is evident that less than half of the reviewed articles developed data visualization software (23 of the 57 articles). In addition, it is notable that among these 23 articles, web applications were developed quantitatively as much as mobile applications, despite the potential challenges of developing a mobile application compared to creating a website. Maybe it is because mobile applications could be more beneficial, especially considering that the target users are mainly livestock farmers. Accessing a mobile application from a cell phone would make it easier to track animals from anywhere.

On the other hand, it is important to consider that, even though it is possible to access a website from a mobile device, these pages must be optimized for portable use, not just on computers. This optimization ensures a smooth and user-friendly experience for farmers who rely on mobile applications for livestock tracking and monitoring.

### 4.7. Feature Integration in the Reviewed Articles

**Figure 8:**
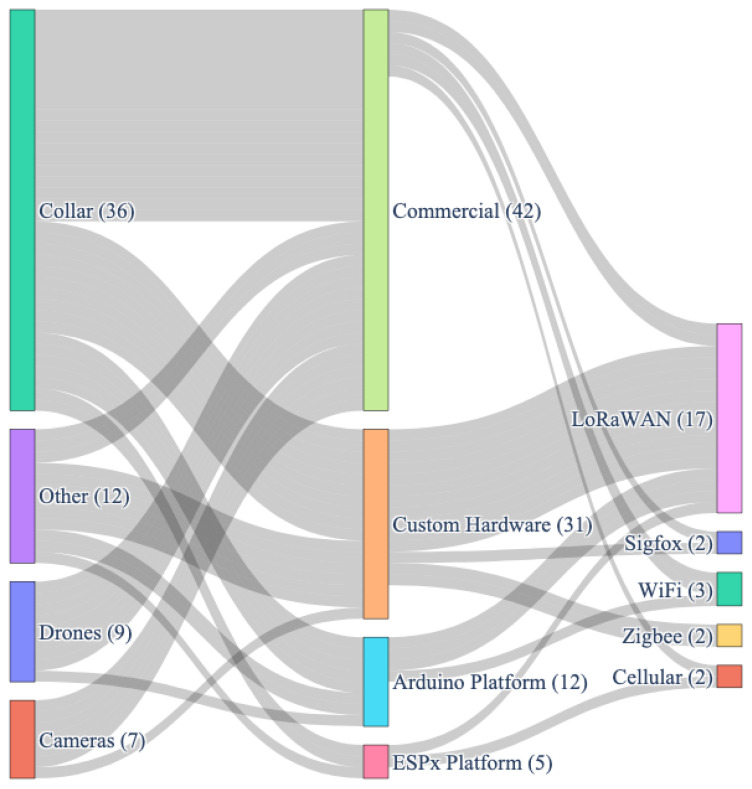
Feature integration flow: solution, device, and wireless communication.

Figure 8 describes the feature selection flow for a solution used in the articles reviewed. It shows the trend of the number of articles selecting a solution (Collar, Drone, Camera, Other), progresses to the type of device, and ends at the wireless communication technology.

It is observed that more than half of the solutions using collars show a tendency to use a commercial device to a customizable one but not a tendency to use a specific communication technology. The solutions that implement a custom hardware choose to select LoRaWAN for wireless communication, with Zigbee in second place. It also shows the tendency of solutions that implement drones and cameras to use commercial equipment; however, it does not rule out the fact that the authors can develop customizable equipment for their solution based on Arduino.

## 5. Solution Selection Strategy

### 5.1. Cattle Tracking

This section aims to provide a comprehensive comparison of various characteristics identified throughout the research process. Four key features are singled out for evaluation: ease of use, cost scalability for large numbers of animals, precision, and invasiveness. These criteria are shown in Table 9. The letters H, M, and L indicate the levels of each characteristic, representing High, Medium, and Low, respectively. The following definitions clarify each attribute:

- **Ease of Use:** This criterion assesses the user-friendliness of a product, emphasizing that the end user does not require any technical expertise. The tracking process should be automatic, ensuring a straightforward and hassle-free experience.
- **Cost Scalability:** The evaluation of cost scalability focuses on whether the financial investment increases proportionally with the number of animals being tracked. This factor is crucial for large-scale livestock operations, where cost efficiency is very important.
- **Precision:** Precision indicates the accuracy and directness of obtaining the animal’s location. A precise tracking solution ensures that the location data are acquired directly from the animal, contributing to the overall reliability of the system.
- **Invasiveness:** Invasiveness refers to the degree to which the tracking product comes into contact with the animal’s body. This factor is essential in considering the impact on the animal’s well-being and comfort during the tracking process.

By systematically evaluating solutions against these identified features, a nuanced understanding of their strengths and limitations will guide the selection process. The goal is to identify a cattle tracking solution that aligns with the specific needs and priorities of the livestock management system, considering factors such as ease of use, scalability, precision, and the level of invasiveness.

In addition to the comparison above, Table 10 provides a detailed comparison of livestock monitoring methods, highlighting their characteristics, strengths, and weaknesses.

#### 5.1.1. Collar

The collar emerges as a precise tracking solution, offering the advantage of incorporating multiple sensors to gather extensive information about the animal’s condition. It is particularly recommended for scenarios with a limited number of animals, where ease of use is crucial for farmers. However, its practicality diminishes when dealing with a large number of animals, as the cost escalates with the need for a collar per animal. Collars are highly user-friendly for farmers requiring minimal intervention, and are well-suited for large expanses of land.

#### 5.1.2. Drone

Drones present a promising alternative for cattle recognition and tracking. They provide a comprehensive view of terrains, potentially reducing overall tracking system costs. Despite their economic feasibility for scenarios with a large number of animals, drones are less precise compared to collars since they rely on artificial intelligence for animal detection rather than obtaining direct location data. Operating a drone is more complex for end-users, requiring piloting skills for effective image and video capture. Drones are recommended for vast expanses, provided they have sufficient range and battery life.

#### 5.1.3. Camera

Cameras are recommended for small areas due to their static nature, resulting in a limited field of vision. They excel in monitoring animal behavior rather than tracking, making them suitable for applications such as disease detection and analyzing mating cycles. They are recommended for monitoring livestock in confined spaces. Cameras are used for detailed observation in controlled environments where animals are not prone to move away from the tracking device.

#### 5.1.4. Definitions of High, Medium, and Low

- Precision:
  − **High:** The system provides location and/or monitoring data with a very high degree of accuracy, presenting a minimal margin of error. This allows for detailed and reliable livestock tracking; suitable for applications requiring precise information.
  − **Medium:** The system offers reasonably accurate data; suitable for general livestock tracking. It may present a larger margin of error compared to high-precision solutions but remains useful for many practical applications.
  − **Low:** The system provides data with a higher margin of error; suitable for basic livestock tracking. It may not meet requirements demanding high accuracy in information.
- Cost:
  − **High:** Implies a significant financial investment in acquiring, implementing, and maintaining the solution. Costs may include advanced devices, specialized communication infrastructure, and possible recurring operational fees.
  − **Medium:** Represents a moderate financial investment. Associated costs are acceptable for many users, although they may require budget considerations for certain operations.
  − **Low:** Involves a reduced financial investment, making the solution economical and accessible for most users. Minimal costs may be due to the use of low-priced components and open technologies.
- Scalability:
  − **High:** The solution has a robust capacity to expand and efficiently manage a large number of devices or animals without performance degradation. It facilitates the integration of new elements into the existing system with minimal additional complexity.
  − **Medium:** The solution allows for expansion to manage a larger number of devices or animals but may require additional infrastructural adjustments, more complex configurations, or increased operational costs.
  − **Low:** The solution presents significant limitations in scaling up and managing an increasing number of devices or animals. Expansion may face technical or logistical challenges and may not be viable without substantial system changes.

### 5.2. Network

In the IoT (Internet of Things) domain, the choice of wireless network technology plays a crucial role in facilitating seamless communication between sensors within a node and the edge server. Given the finite battery life of IoT devices, prioritizing low energy consumption is essential to extend battery longevity. Various optimization techniques such as data compression, duty cycling, efficient routing, mobility, and approximate computing are commonly employed. In addition, implementation cost becomes an important factor when comparing proprietary and off-the-shelf technologies.

#### 5.2.1. LoRaWAN

LoRaWAN stands out as a highly efficient wireless networking option, achieves significant energy savings compared to Zigbee, cellular, and Wi-Fi networks. By leveraging transmission technologies such as LoRa, LoRaWAN can achieve remarkable reductions in power consumption, potentially up to 60%. In addition, LoRaWAN’s efficiency increases significantly in unobstructed environments, where it can provide reliable communication over long distances. It is, therefore, recommended for use in collar-based tracking solutions, where energy efficiency and extended coverage are paramount considerations.

#### 5.2.2. Wi-Fi

Wi-Fi networks are predominantly utilized in indoor environments, providing reliable wireless connectivity within confined spaces. These networks are particularly well-suited for IoT applications deployed in indoor settings, such as surveillance cameras or environmental monitoring systems. IoT devices equipped with Wi-Fi capabilities can seamlessly transmit data over local area networks, enabling real-time monitoring and data collection. Consequently, Wi-Fi networks are ideal for applications where IoT devices operate within the confines of indoor spaces and rely on Wi-Fi connectivity for data transmission.

#### 5.2.3. Zigbee

Zigbee has greater coverage than Wi-Fi and less than LoRa, and its advantages of use are that devices are accessible and easily programmable. Unlike Lora, Zigbee increases the transmission rate and is, therefore, recommended for sending data from several nodes or a gateway to a device that allows for internet connection and, thus, uploading the data to the cloud.

#### 5.2.4. Sigfox

Sigfox is ideal for very large areas of terrain (>10 km) and is recommended if transmission rates are in the hundreds of bps range [72]. Being a proprietary technology, a rental cost must be paid for the services provided by the radio stations (collecting and sending data), and it must also be taken into account that the coverage is different depending on the area and region in the world. For Sigfox, there are suppliers that deliver ready-to-use devices, which avoids the design and construction of the entire solution.

#### 5.2.5. Cellular

Cellular networks have almost no coverage in rural areas, and for large extensions, GPRS (2G) networks are used for data transmission. They are suitable for urban environments, and a high cost must be considered because the construction of devices that connect to these networks considers a wireless connection module, the cost of connection to the cellular network, and the data plan. For animal monitoring, cellular networks are recommended to connect the device directly to the cloud without the need to go through a gateway.

#### 5.2.6. Others

Others mainly consider the integrated wireless communication provided by some equipment such as drones and commercial RF modules. It is recommended to use these when the chosen solution is the use of drones; you can take advantage of the connection that the drone has with its remote control and, if possible, make the modification for this drone to send the required data (e.g., location, sensor data, etc.). The range of this connection is limited by the integrated equipment of the drone. Commercial RF modules using other technologies are also used; they are more adaptable to a specific solution but require more testing for connection, stability, range, and power consumption than other standardized technologies.

### 5.3. Sensors

After selecting the most suitable device (applicable to both collars and drones), an essential consideration is the number of measurements desired, including location, individual identification, animal biometrics, physical activity, and environmental metrics. Increasing the number of parameters to measure typically involves using multiple sensors, significantly increasing the weight placed on the device (or on the animal). However, with advancements in nanotechnology and sensor miniaturization, this is not a highly limiting factor for some cases.

Battery life is also affected by the power consumption of one or multiple sensors and the operating time. For instance, sensors such as GPS, accelerometers, and heart rate monitors have varying power consumption levels. The duration of operation depends on how long the sensors need to be active for and whether they are continuously monitoring or intermittently transmitting data.

#### 5.3.1. GPS

The Global Positioning System (GPS) sensor is indispensable, particularly in collar-based tracking systems, as it provides precise location data for the animals being monitored. This information is crucial for accurately tracking the movement and behavior of individual animals. However, in the case of cameras, the inclusion of a GPS may not be necessary since their focus is typically on capturing visual data rather than obtaining precise location coordinates. Similarly, while drones may incorporate a GPS, its utility may be limited as drones often prioritize identifying multiple animals simultaneously rather than pinpointing individual locations.

#### 5.3.2. Temperature

Temperature sensors play a vital role in collar-based tracking systems, offering valuable insights into the health and well-being of the monitored animals. By monitoring changes in body temperature, farmers can detect signs of illness or distress early on, allowing for prompt intervention and care. However, temperature sensors are not applicable to drones or cameras, as their primary function is to capture visual data rather than physiological measurements.

#### 5.3.3. Humidity

In collar-based tracking systems, humidity sensors can provide additional data points that help farmers monitor various aspects of animal behavior and physiology. For example, monitoring humidity levels can aid in tracking mating cycles or identifying environmental conditions that may affect animal health and well-being. However, humidity sensors are not suitable for integration into drones or cameras, as their functionality is limited to collar-based tracking systems.

#### 5.3.4. Accelerometer

Accelerometers are versatile sensors that can be employed in various tracking systems to recognize and monitor animal activities. By detecting changes in acceleration and movement patterns, accelerometers enable the identification of specific behaviors such as grazing, walking, or resting. This information is valuable for understanding animal behavior patterns and detecting deviations that may indicate health issues or abnormal activity. Additionally, accelerometers are considered a cost-effective and energy-efficient solution, making them widely adopted in tracking systems.

#### 5.3.5. Buzzer

Sound devices can also be added to these tracking systems in cattle, which become useful in cases of integration with collars. First of all, they can be utilized in guiding the cattle within a virtual fence due to automatic sound emission that can help in keeping them away from the boundary. Secondly, in mislaid animals or a zone where the visibility is poor, the buzzer can be remotely turned on by the owner from his mobile device to generate a sound for locating the stray animal.

## 6. Future Trends and Directions

Currently, there is a clear trend towards the application of artificial intelligence (AI), including techniques such as machine learning and neural networks, in almost every aspect of our daily lives, from virtual assistants to personalized recommendations in entertainment platforms. In the realm of Internet of Things (IoT) and Smart Farming, these technologies are redefining traditional processes through automation and predictive analytics [73]. AI is being used to monitor and optimize resource use, improve animal welfare, and predict disease in livestock by integrating advanced sensors and processing large volumes of data in real time. This article briefly discusses some of these applications; however, it should be clarified that the technology trend points towards the implementation of automated and artificial intelligence systems. These applications not only increase operational efficiency, but also enable more accurate and faster decision-making based on patterns and predictions generated by machine learning algorithms.

Artificial intelligence and machine learning play a crucial role in interpreting large volumes of data from sensors and portable devices. These systems improve the ability to predict health problems based on historical data and detected livestock behaviors, alerting farmers to potential diseases before visible symptoms occur. As AI evolves, systems will be able to learn from normal livestock behavior and detect subtle deviations that indicate stress or disease; the integration of advanced technologies such as computer vision and artificial intelligence (AI) is leading to this evolution. Computer vision allows for visual identification of livestock, monitoring of their behavior, and early detection of disease by analyzing images and videos. The use of deep learning algorithms and neural networks, such as YOLO (You Only Look Once), facilitates real-time tracking, and their implementation with drones and fixed cameras allows for large-scale surveillance without constant human intervention.

Automation in precision agriculture is reducing production costs by minimizing the need for intensive labor. Wearable sensors and remote monitoring systems enable continuous livestock tracking at relatively low costs, with devices such as smart collars collecting and transmitting data through IoT networks. This technology makes it possible to manage large numbers of livestock efficiently, reducing losses due to disease or theft. Integrating data from multiple sources creates a more complete picture of the health status of livestock, enabling more efficient and accurate monitoring, with automatic alerts and rapid response systems.

It is anticipated that interest in livestock tracking solutions will continue to grow globally, driven by the need to meet world food demand and improve sustainability in livestock farming. Emerging regions in this field, such as Africa and South America, could experience an increase in research as access to technologies and international collaborations increases.

In the future, these technologies are expected to be fully integrated with Internet of Things (IoT) platforms, enabling more efficient and sustainable livestock management. Advanced automation, including the use of autonomous robots and drones, will optimize processes such as feeding, milking, and health monitoring. These advances will help improve animal welfare, increase productivity, and meet the growing demands of the agricultural sector.

## 7. Conclusions

In the field of livestock tracking solutions, collars are emerging as the preferred option due to their versatility in integrating various sensors, facilitating both location tracking and monitoring of vital parameters. However, despite their high efficiency, the scalability of collar-based solutions poses a major challenge, as costs increase proportionally to the size of the herd. This underlines the need to carefully consider budget constraints when implementing monitoring systems on large livestock farms.

Alternatively, static cameras and drones offer more economical options for livestock monitoring, albeit with their own limitations. Drones, although less accurate, offer a complete aerial view, which can reduce the overall costs of the tracking system. However, their operational complexity and short battery life pose problems, especially in remote or difficult environments. Cameras, while cost-effective, are more suitable for monitoring animal behavior in confined spaces than for tracking animal movements over large areas, but their accuracy is lower.

Commercial devices, such as GPS loggers and collars, are chosen over custom boards, Arduino, or ESP because of their ease of implementation and use, but not because of their price, which is much higher than making a collar. However, it is noted that the creation of custom boards reflects a broader trend towards customizable and adaptable tracking solutions because they allow for the integration of a wide variety of sensors. This adaptability is crucial to address the diverse needs and challenges faced in real-life livestock management scenarios, where factors such as terrain, climate, and animal behavior vary and is an important parameter to measure.

Real-world testing is an essential component of solution development, as it allows researchers and practitioners to evaluate the effectiveness and reliability of proposed tracking systems in practical situations. These tests provide valuable information on the performance of devices in different environmental conditions, as well as their ability to operate autonomously and withstand the rigors of daily farm use.

In other words, the upward trend in livestock tracking is moving toward integrated and effective solutions through innovation and commitment to practical application. Overcoming challenges, particularly in terms of cost-effectiveness and scalability, will require further research and development to ensure viable technologies emerge for accurate and efficient tracking across diverse agricultural landscapes. This review analyzes various scenarios and state-of-the-art technologies, providing a solution selection strategy to help readers identify crucial factors when deciding which solution best fits their needs. These technologies demonstrate considerable differences in how cattle are monitored and tracked. Collars remain one of the most trusted solutions due to their precision. However, with the rapid development of machine learning and computer vision, more drone-based solutions are expected to be explored, as they offer a cost-effective way to cover larger areas.

## Figures and Tables

**Table 1:** Scientific database search results.

Scientific Database	Search String	Results
ACM	“cattle tracking” “cattle geolocalization” “livestock tracking” “livestock geolocalization”	54
IEEE	(cattle tracking) OR (cattle geolocalization) OR (livestock tracking) OR (livestock geolocalization)	70
ScienceDirect	“SMART FARMING” AND (“CATTLE TRACKING” OR “CATTLE GEOLOCALIZATION” OR “LIVESTOCK TRACKING” OR “LIVESTOCK GEOLOCALIZATION”)	10
Scopus	“cattle tracking” OR “cattle geolocalization” OR “livestock tracking” OR “livestock geolocalization”	60

**Table 2:** Solution classification.

Solution	Reference	Quantity
Collar	[6,11,12,13,14,15,16,17,18,19,20,21,22,23,24,25,26,27,28,29,30,31,32,33,34,35,36,37,38,39,40,41,42,43,44,45]	36
Camera	[12,46,47,48,49,50,51,52,53,54,55,56,57]	13
Drone	[21,27,42,49,50,52,54,55,56,58]	10
Other	[18,58,59,60,61,62,63,64,65]	9

**Table 3:** CPU device classification.

Device	Reference	Quantity
Custom	[12,13,18,24,25,26,28,31,32,39,59,61,62,63,65]	15
Arduino	[15,16,23,30,38,58,67]	7
ESPx	[17,20,67]	3
Commercial	[14,19,21,29,33,35,36,40,41,42,43,44,45,49,50,52,54,55,56,60]	20

**Table 4:** Sensor and actuator classification.

Sensor	Reference	Quantity
GPS	[6,12,13,14,15,17,18,20,21,22,24,25,26,28,29,30,31,32,33,34,35,36,38,39,41,42,43,44,50,54,56,58,59,60,65,67]	36
Humidity	[17,25]	2
Temperature	[17,18,20,25,32,34,39,61]	8
Pulse	[17,18]	2
Accelerometer	[18,19,23,28,30,31,32,36,39,41,44,45,61,63,64]	15
Other	[12,13,15,17,18,34,39,43,45,58,61,67]	12

**Table 5:** Power supply.

Power Supply	Reference	Quantity
Rechargeable battery	[6,12,13,16,21,22,23,25,26,27,28,30,32,33,35,36,38,39,40,41,43,44,45,49,50,52,54,55,56,60,61,62,63,64,65]	35
Solar panel	[13,21,25,26,61]	5

**Table 6:** Wireless communication technology.

Technology	Reference	Quantity
LoRaWAN	[6,12,13,15,16,18,20,25,26,28,30,31,32,34,39,58,59,61]	18
Zigbee	[24,61]	2
Sigfox	[22,65]	2
Cellular	[11,17]	2
Wi-Fi	[50,54]	2
Other	[19,33,40,45,49,54,62,64,67]	9

**Table 7:** Software to visualize information.

Software	Reference	Quantity
Web application	[13,20,22,25,26,28,34,39,65]	9
Mobile application	[16,25,28,34,39,49,61,63,67]	9
Desktop application	[18,33,43,50,59,60]	6
Other	[11,30,62]	3

**Table 8:** Level of implementation.

Level	Reference	Quantity
Simulated	[6,11,17,18,20,21,23,24,27,31,42,62,63,67]	14
Real	[12,13,14,16,19,22,25,26,28,29,30,32,33,34,35,36,37,38,39,40,41,43,44,45,46,47,48,49,50,51,52,53,54,55,56,57,59,60,64,65]	40
Untested	[15,58,61]	3

**Table 9:** Characteristic comparison of cattle tracking methods.

Characteristic	Drone	Collar	Camera
Ease of Use	M	H	H
Cost Scalability	H	L	M
Precision	M	H	L
Invasiveness	L	M	L

H, M, and L indicate High, Medium, and Low, respectively.

**Table 10:** Comparison of Livestock Monitoring Methods.

Method	Characteristics	Strengths and Weaknesses
Collars	Precision: High Cost: Low to Medium Scalability: High	Strengths: Real-time tracking of livestock location [59]. Prevention of theft and loss, facilitating quick recovery [11]. Monitoring of health and behavior, including vital signs and activity [14]. Easy scalability for large herds [15]. Affordable costs due to the use of technologies like LoRaWAN, SigFox, and low-cost commercial components [67]. Customization and adaptability owing to the use of open hardware and software [16]. Integration with IoT platforms and mobile applications for alerts and data management [13]. Weaknesses: Dependence on communication infrastructure such as GSM, LoRaWAN, and SigFox, which may be limited in remote rural areas [46]. Limited battery life, especially with multiple sensors or frequent transmissions [12]. Requires regular maintenance and possible technical updates [11]. Necessity of technical knowledge for implementation and configuration in some cases [14]. Limitations in data transmission rates and potential interference in certain frequency bands [13].
Drones	Precision: High Cost: High Scalability: Medium	Strengths: Deployment optimization using clustering algorithms like K-Means and DBSCAN [6]. Adaptability in response to animal movement [6]. Use of convolutional neural networks for efficient detection and counting [54]. Possibility to incorporate additional sensors [23]. Integration with advanced technologies like blockchain and IPFS [50]. Weaknesses: Dependence on devices on animals, implying additional costs [24]. Technical complexity and high hardware requirements [53]. Operational limitations due to battery life [6]. Dependence on data accuracy and image quality [54]. Need for reliable communication infrastructure [50].
Cameras	Precision: High Cost: Medium Scalability: High	Strengths: Accurate detection and tracking with advanced techniques [47]. Real-time processing with high speed [47]. Non-intrusive monitoring without the need for devices on animals [18]. Improved handling of variations and occlusions in complex environments [28]. Weaknesses: Dependence on image quality affected by external conditions [63]. Requires powerful hardware for real-time processing [55]. Limitations in extensive areas or uneven terrains [64]. Need for installation and maintenance of infrastructure [18].

## Abbreviations

The following abbreviations are used in this manuscript:

IoT	Internet of Things
RFID	Radio Frequency Identification
GPS	Global Positioning System
CNN	Convolutional Neural Network
YOLO	You Only Look Once
UAV	Unmanned Aerial Vehicle
ToA	Time of Arrival
pH	Potential of Hydrogen
LoRa	Long Range
LoRaWAN	LoRa Wide Area Network
ISM	Industrial, Scientific, and Medical
PASS	Pastoral Animals Shepherd System
CSMA	Carrier Sense Multiple Access
AI	Artificial Intelligence
